# Subcutaneous metastasis – the first sign of hepatocellular carcinoma in chronic virus C hepatitis patient – Case report

**Published:** 2015

**Authors:** I Ciortescu, A Rotariu, DM Cozma, D Negru, R Livadariu, D Diaconu

**Affiliations:** * ”Gr. T. Popa” University of Medicine and Pharmacy, Iaşi, Romania; ** Gastroenterology and Hepatology Centre, “Sf. Spiridon” Hospital, Iaşi, Romania; *** 3rd Surgical Clinic, “Sf. Spiridon” University Hospital, Iaşi, Romania; **** Medical Imaging Department, “Sf. Spiridon” Hospital, Iaşi, Romania

**Keywords:** hepatocellular carcinoma, subcutaneous tumor, chronic HCV hepatitis, cirrhosis, extrahepatic metastasis

## Abstract

Hepatocellular carcinomas make up 90% of primary liver cancers. The association between the hepatic carcinoma and virus B and C infection has been already proven. Hepatocellular carcinoma develops, in most cases, on a background of cirrhosis and rarely in hepatitis. The case we have chosen to report distinguishes itself due to the unusual extra-hepatic metastatic location of a hepatocellular carcinoma in a patient with Chronic HCV hepatitis.

## Case report

We report the case of a 62-year-old male patient, H.V., from rural area, who was admitted in our clinic in March 2012, complaining of general asthenia and the occurrence of a subcutaneous painless mass located in the supero-external quadrant of the right breast, about a month earlier, which had been progressively increasing in size.

The patient’s medical history included chronic virus C hepatitis since 2008, laparoscopic cholecystectomy in February 2011. A hepatic biopsy was performed during this procedure and the anatomopathological exam revealed mild inflammation (score 2) and minimal fibrosis (F1). Considering the hepatitis stage of the disease and the presence of viral replication (RNA-HCV 561.964UI/ ml), the initiation of an antiviral peginterferon and ribavirin therapy was recommended and the patient was put on a waiting list.

Physical examination was unremarkable, except for an 80/ 90mm relatively well-delimited and partially mobile tumor located in the supero-external quadrant of the right breast, with normal overlying tegument. No local-regional adenopathies were identified.

Laboratory investigations: hepatocytolysis (ALT=85U/ L, AST=119U/ L), normal complete blood count, no coagulation disorders, AFP=26.2ng/ ml (normal range: 0-5.5ng/ ml). 

Abdominal ultrasound: homogeneous liver, no signs of portal hypertension, normal spleen, no ascites. Upper gastrointestinal endoscopy: no esophageal and gastric varices. Fibrosis assessed by transient elastometry (FIBROSCAN) – minimal fibrosis F1.

Chest X-ray in P/ A incidence: an opaque mass of about 10cm with right basal location, well delimited, of average intensity and osteolysis of C4-C5 anterior right arcs. In lateral view, the mass projected over the heart.

**Fig. 1:**
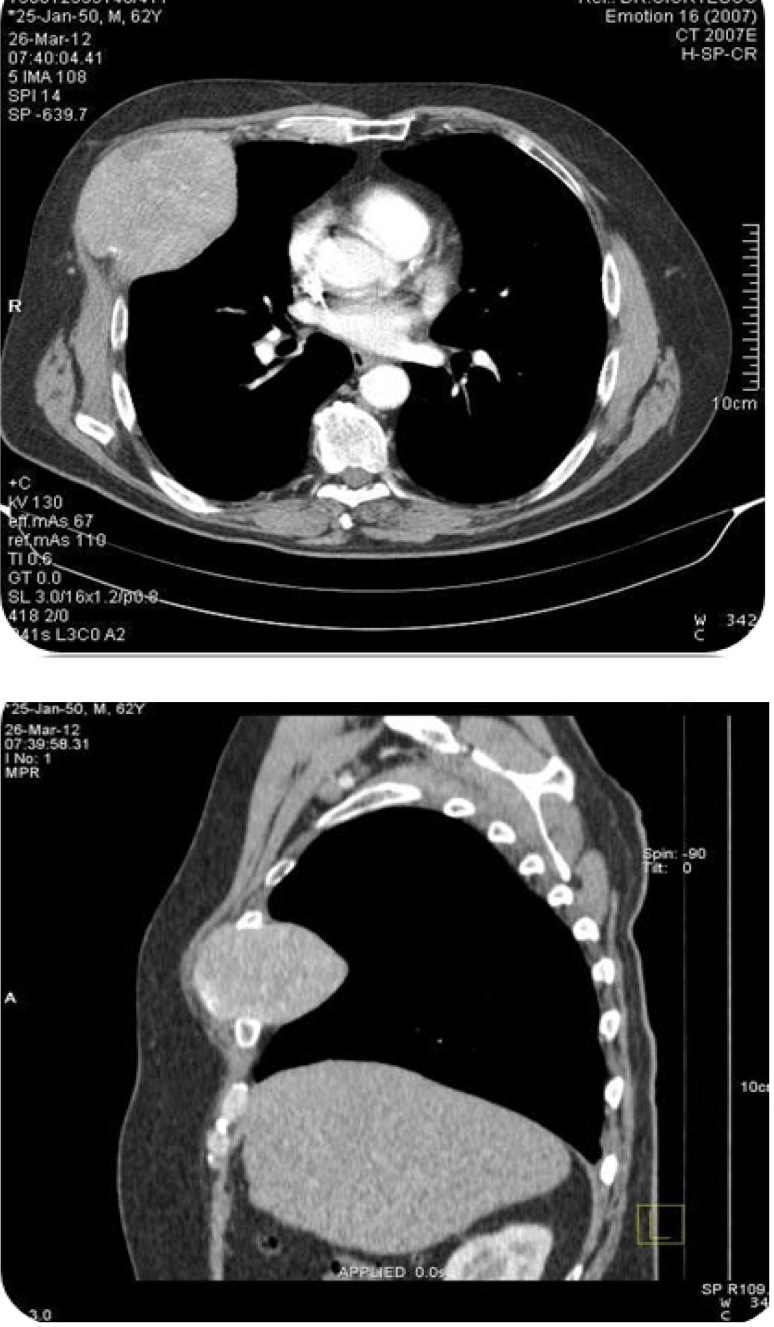
Thoracic computerized tomography showing a lesion of 7,9 cm x 9,5 cm in diameter on the right anterolateral chest wall

The contrast-enhanced computed tomography of the thorax revealed: relatively well-delimited solid expanding mass, with intense and non-homogeneous contrast uptake, with inner necrosis areas and 79/95/71mm sizes (AP/T/CC), located in the right hemithorax between ribs 4 and 5. The mass caused complete osseous lysis of the anterior arch of rib 5, lysis of the lower border of the anterior arch of rib 4 and minimum lysis of the upper border of the anterior arch of rib 6. The mass compressed the right pectoralis major muscle, without invading it (**Fig. 1**). No expanding masses were detected in the lung parenchyma. Normal native liver examination.

At that point, the diagnosis of subcutaneous thoracic tumor (possibly sarcoma) and chronic HCV hepatitis motivated the transfer of the patient in the surgery department. The surgery revealed a tumor mass with pseudo-capsule located under the right pectoralis major muscle, which, when cut, exhibited necrotic and hemorrhagic tumor tissue. The extemporaneous examination of the sample revealed an epithelioid-like tumor proliferation, with large cells, voluminous nuclei, rich nucleoli and cytoplasm, morphological appearance that might have revealed a malignant melanoma. Due to considerable bleeding, the complete mass excision was not possible. The postoperative evolution was positive, with no local bleeding or pneumothorax.

**Fig. 2:**
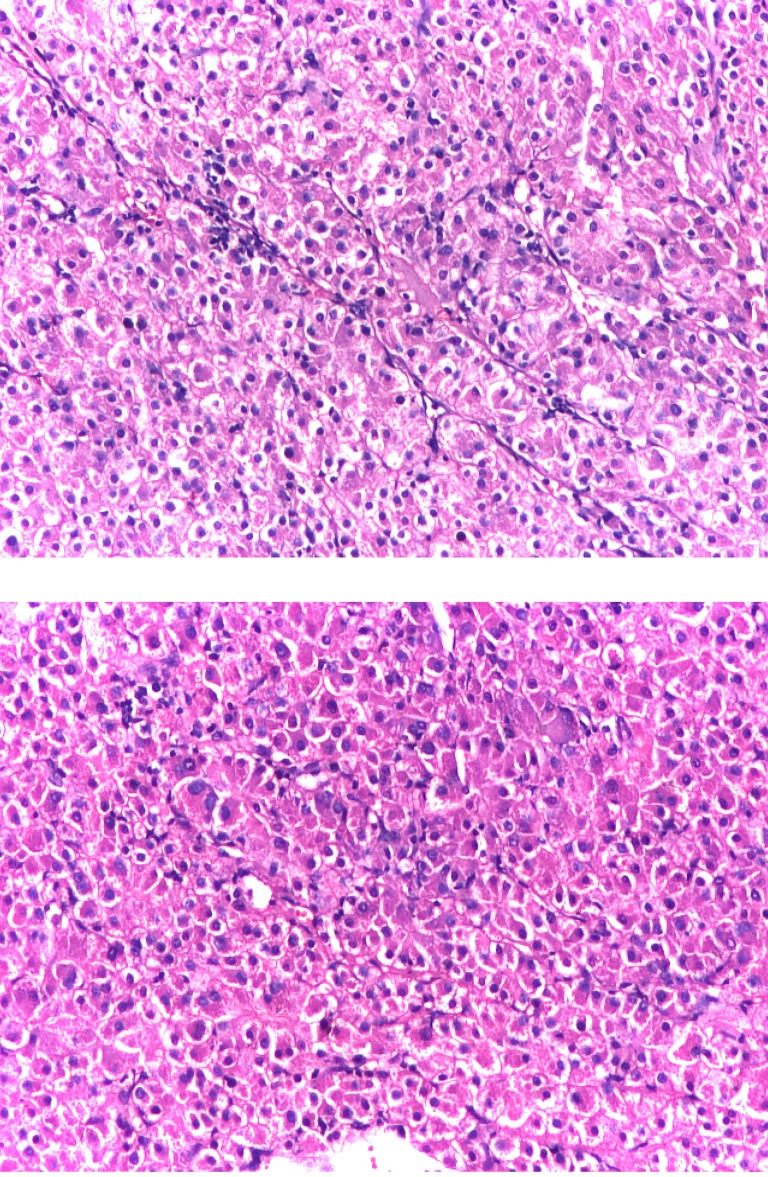
Microscopic view revealed moderately differentiated hepatocellular carcinoma (H&E, x10)

The histopathological exam of the tumor specimen revealed: epithelial proliferation with solid or macrotrabecular architecture, fine sinusoidal-like vessels, tube-shaped structures, acinus whose lumen exhibited eosinophilic corpuscular secretion and very rarely yellow-brown-greenish bile pigment. The anatomopathological diagnosis was metastases of moderately differentiated, solid or trabecular, hepatocellular carcinoma (**Fig. 2**). 

**Fig. 3:**
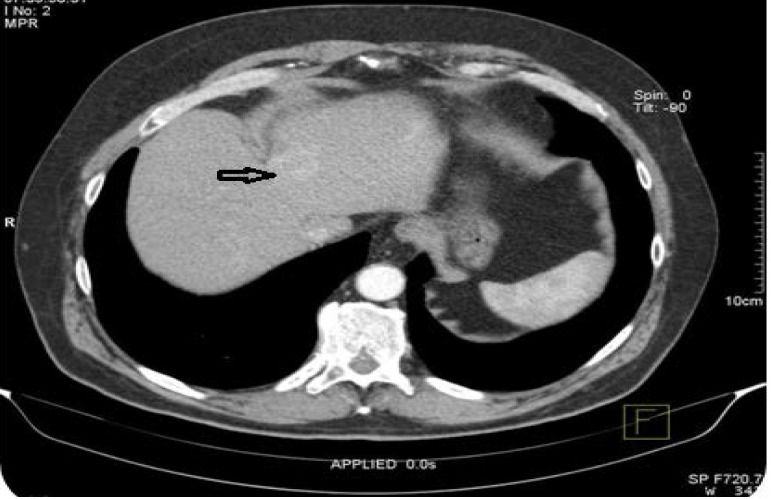
Abdominal computed tomography showing a solid hepatic mass of 3cm x 2,8cm occupying the border between segments II and III (see black arrow)

A new computed tomography scan of the abdomen was performed one month after the first assessment, which revealed a solid hepatic expanding subcapsular mass of 30/ 28mm, located on the border between segments II and III, with native iso-hypodense appearance, with intense and non-homogeneous contrast uptake in arterial time, which becomes hypodense in venous and late times (**Fig. 3**). 

The final diagnosis in this case was Hepatocellular carcinoma BCLC stage C. Subcutaneous metastasis. Chronic HCV hepatitis. The patient was referred to the oncology department and in June 2012, a sorafenib therapy was started. 

## Discussions

This is the case of a 62-year-old patient known with chronic HCV infection since 2008, in whom the hepatocellular carcinoma diagnosis was set in the hepatitis stage of his liver disease, given the occurrence of a subcutaneous metastasis.

The patient was diagnosed with chronic HCV infection by his general practitioner at the age of 58, further to routine medical tests that detected a moderate hepatic cytolysis syndrome. The patient’s anamnesis identified no major HCV infection risk factors (no transfusions, no surgical procedures, no invasive procedures, etc.). The patient has been married for over 20 years, and his wife had no HCV infection. In this case, we were unable to determine the probable date of infection contacting.

In February 2011, when the patient came to our clinic for an uncomplicated biliary colic, the hepatic condition was evaluated and the viral load was determined. According to the clinical, biochemical and imaging exams, the diagnosis established was Chronic HCV hepatitis with mild viral load (RNA-HCV 561.964UI/ ml). The patient was proposed for antiviral peginterferon and ribavirin therapy. The liver biopsy performed during the laparoscopic cholecystectomy confirmed the hepatitis stage of the hepatic HCV condition (Ishak necroinflammatory score: mild inflammation-score 2, minimal fibrosis F1). In these conditions, we thought that the subcutaneous tumor had no connection with the hepatic disease, because the risk of liver cancer with metastasis in a patient with chronic HCV hepatitis is low (4.8%) [**1**-**4**]. Also, we interpreted the slightly high AFP value (AFP=26.2ng/ mL) as triggered by hepatic cytolysis. For these reasons, the first computed tomography was performed with contrast medium only for the thorax, and not for the liver.

The computed tomography of the chest revealed no pulmonary lesions, but only osseous lysis, which was interpreted as a possible local tumor invasion of the ribs.

Pulmonary and osseous metastases can accompany hepatocellular carcinoma in a relatively high percentage (47% and 37%, respectively) [**5**], but there are few cases of cutaneous metastases by hepatic cancer described in literature (2.7% in cirrhotic patients, no case of non-cirrhotic patients) [**6**]. No case of subcutaneous metastasis by hepatocellular carcinoma was reported in literature. 

The second computed tomography of the liver was made, in the light of the anatomopathological exam result, with serious clinical suspicion and one month later. Considering that the diameter at the time of the imaging diagnosis setting was 30/ 28mm, it is highly unlikely that the sizes of the liver mass would have been below the detection limits at the first examination.

In conclusion, the risk of liver cancer is real in patients with chronic HCV hepatitis. Ultrasound follow-up by experts, using high performance devices, at short time intervals (3 months) would be ideal for an early hepatocellular cancer diagnosis in patients with HCV hepatitis. In Romania, this desideratum is virtually impossible, especially due to objective reasons (equipment).

Even if we have detected the hepatic tumor before the diagnosis of hepatocellular carcinoma metastasis in our case, our medical conduct would have been the same. Wall chest tumor biopsy was compulsory and much more accessible than liver mass biopsy.

Another issue to discuss was the seat of the hepatocellular carcinoma metastasis. One possibility would have been pulmonary metastasis with osseous and subcutaneous extension. This assumption was invalidated by the absence of any pulmonary lesions at the computed tomography scan. One could not exclude osseous metastasis spreading in the neighboring tissues either, considering that hepatocellular carcinoma often has metastasis in the bones. We believe that the metastasis was subcutaneous, in the connective tissue, with osseous invasion.

The patient did not receive antiviral therapy (peginterferon and ribavirin), being on the waiting list at the time of subcutaneous metastasis occurrence. Considering the low viral replication level (RNA-HCV 561.964UI/ ml) and the hepatitis stage of the disease, it is rather unlikely that viral replication inhibition would have excluded hepatocellular carcinoma occurrence. In this case, we believe that the influence of other factors (diet, alcohol intake, and genetics) was greater than that of HCV infection in the carcinogenesis process.

## Conclusions

Virus C hepatitis infection is a risk factor for liver cancer. Hepatocellular carcinoma may also develop on a non-cirrhotic patient. The first sign of liver cancer may be an extra-hepatic metastasis.

**Conflict of interests**

Nothing to declare
